# Energy absorption buildup factors of human organs and tissues at energies and penetration depths relevant for radiotherapy and diagnostics

**DOI:** 10.1120/jacmp.v12i4.3557

**Published:** 2011-11-15

**Authors:** S. R. Manohara, S. M. Hanagodimath, L. Gerward

**Affiliations:** ^1^ Department of Physics Siddaganga Institute of Technology Tumkur‐572 103 Karnataka India; ^2^ Department of Physics Gulbarga University Gulbarga‐585 106 Karnataka India; ^3^ Department of Physics Technical University of Denmark DK‐2800 Lyngby Denmark

**Keywords:** gamma radiation, interaction of gamma/X‐ray radiation with matter, buildup factor, effective atomic number, tissue equivalence, GP fitting formula, human organs and tissues

## Abstract

Energy absorption geometric progression (GP) fitting parameters and the corresponding buildup factors have been computed for human organs and tissues, such as adipose tissue, blood (whole), cortical bone, brain (grey/white matter), breast tissue, eye lens, lung tissue, skeletal muscle, ovary, testis, soft tissue, and soft tissue (4‐component), for the photon energy range 0.015–15 MeV and for penetration depths up to 40 mfp (mean free path). The chemical composition of human organs and tissues is seen to influence the energy absorption buildup factors. It is also found that the buildup factor of human organs and tissues changes significantly with the change of incident photon energy and effective atomic number, $Z_{\text{eff}}$. These changes are due to the dominance of different photon interaction processes in different energy regions and different chemical compositions of human organs and tissues.

With the proper knowledge of buildup factors of human organs and tissues, energy absorption in the human body can be carefully controlled. The present results will help in estimating safe dose levels for radiotherapy patients and also useful in diagnostics and dosimetry. The tissue‐equivalent materials for skeletal muscle, adipose tissue, cortical bone, and lung tissue are also discussed. It is observed that water and MS20 are good tissue equivalent materials for skeletal muscle in the extended energy range.

PACS numbers: 32.80‐t, 87.53‐j, 78.70‐g, 78.70‐Ck

## I. INTRODUCTION

The biological effect of ionizing radiation on human beings depends on absorbed dose, type of radiation (alpha, beta, gamma, etc.), energy, and organs irradiated. Photons entering the body not only loose energy and are finally absorbed, but also they give rise to new photons by multiple scattering. The magnitude of the latter effect can be estimated by the so‐called buildup factor. The buildup factor is a multiplicative factor used to get the corrected response to uncollided photons by including the contribution of scattered photons. The buildup factor depends on the atomic number of the absorbing medium, the energy of the gamma rays, and the penetration depth, as well as the shape of the radiation source and the medium. Buildup factors of point isotropic sources are widely used for dose estimations, and in the design of gamma‐ray shields and phantoms. The *energy absorption buildup factor* ^(^ ^1^ ^)^ is defined as the photon buildup factor in which the quantity of interest is the absorbed or deposited energy in the medium considered. Harima^(^ ^1^ ^)^ has given a detailed review on calculations and applications of buildup factors.

The American Nuclear Society Standard Committee working group^(^ ^2^ ^)^ has developed a set of gamma‐ray point isotropic source buildup factors as a standard reference database for 23 elements in the range Z=4−92 and three compounds or mixtures — namely, air, water and concrete — for use in shielding calculations. The ANSI/ANS‐6.4.3‐1991 standard^(^ ^2^ ^)^ for buildup factors has been administratively withdrawn, but work is in progress for updating this standard which is much used.^(^ ^3^ ^,^ ^4^ ^)^ For the time being there are no new reference data for buildup factors. Meanwhile, it should be all right to use the 1991 standard,^(^ ^2^ ^)^ since the possible discrepancies are expected to be small for the low‐Z materials (see section III.B.4 below).^(^ ^3^ ^,^ ^4^ ^)^

Tissue equivalence describes the property of the material to respond to radiation in the same way as human tissue. Phantoms made with tissue substitutes are widely used in medicine, radiation therapy, diagnostic radiology, radiation protection, and radiobiology to calibrate radiation detector systems and for depth‐dose estimates. In the present study, we will define tissue equivalence in terms of the effective atomic number, $Z_{\text{eff}}$, which represents a weighted average of the number of electrons per atom in a multi‐element material.^(^ ^5^ ^)^ Early calculations of $Z_{\text{eff}}$ were based on parameterization of the photon interaction cross section by fitting data over limited ranges of energy and atomic number. Today, accurate databases and interpolation programs, such as WinXCom,^(^ ^6^ ^,^ ^7^ ^)^ have made it possible to calculate $Z_{\text{eff}}$ with much improved accuracy and information content over wide ranges of photon energy, and for all types of materials.^(^ ^5^ ^)^

Gamma rays and X‐rays are commonly used for diagnostic (nuclear medicine, CT (computed tomography) scanning, radiography, mammography, etc.) and for treatment (radiotherapy, gamma knife radiosurgery, radiology, etc.) of many diseases. As a consequence, various human organs and tissues will be exposed to X‐rays and gamma rays. Hence it is necessary to have the knowledge about how these radiations interact with the human body. Because when photons enter the body, they degrade their energy and build up inside the body, giving rise to secondary radiation which can be estimated by “buildup factor”.

When various human organs and tissues are exposed to X‐rays and gamma rays, then the maximum radiation dose to the tissue may not be at its surface but somewhere inside, due to the buildup of degraded photons by multiple scattering within the tissue. Recognizing the importance of buildup of such scattered photons, an attempt has been made to compute the energy absorption buildup factors for some human organs and tissues. Recently, Kurudirek and Özdemir^(^ ^8^ ^)^ have reported the buildup factors for some polymers and tissue substitute materials. However, such information is lacking as far as mixtures and compounds are concerned. Hence, there is a need for gamma‐ray buildup factors of low‐Z complex materials, such as human organs and tissues, in diagnostics, dosimetry, and radiation therapy for absorbed dose estimations. This prompted us to take up the study on the buildup of photons in some human organs and tissues, for which the buildup factor data cannot be found in any compilation or tabulation. With proper knowledge of buildup factors of human organs and tissues, energy absorption in the human body can be carefully controlled. The results of the present paper will also help in estimating safe dose levels for radiotherapy patients and also will be useful in dosimetry and diagnostics.

In the present work, we have calculated geometric progression (GP) fitting parameters and energy absorption buildup factors for human organs and tissues, such as adipose tissue, blood (whole), cortical bone, brain (grey/white matter), breast tissue, eye lens, lung tissue, skeletal muscle, ovary, testis, soft tissue, and soft tissue (4‐component) by using the ANSI/ ANS‐6.4.3‐1991 standard data and the GP fitting formula of Harima et al.^(^ ^9^ ^)^ The calculations have been performed for exposures in the radiotheraptic/diagnostic for gamma (or X‐ray) energy range 15 keV–15 MeV up to penetration depths of 40 mfp (mean free path). The generated buildup factor data is studied as a function of incident photon energy, chemical composition, and effective atomic number, $Z_{\text{eff}}$. The tissue‐equivalent materials for skeletal muscle, adipose tissue, cortical bone, and lung tissue are also discussed. It is observed that water and MS20 are excellent tissue‐equivalent materials for skeletal muscle in the extended energy range. The data are useful for many applications to medical physicists, especially in radiation therapy and dosimetry, for the construction of phantoms using tissue‐equivalent materials.

## II. MATERIALS AND METHODS

The chemical compositions of the human organs and tissues are given in Table 1. These data have been taken from literature.^(^ ^10^ ^,^ ^11^ ^)^ The computations have been carried out in three steps, as follows:

**Table 1:** Elemental composition of human organs and tissues^(^ ^10^ ^,^ ^11^ ^)^ and approximate values of $E_{\text{pe}}$, $E_{\text{pp}}$, and $E_{\text{peak}}$.

*S.N*	*Human Organs and Tissues*	*Composition (element: fraction by weight)*	$E_{\text{pe}}$ *(MeV)*	$E_{\text{pp}}$ *(MeV)*	$E_{\text{peak}}$ *(MeV)*
1.	Adipose tissue	*H*: 0.114, *C*: 0.598, *N*: 0.007, *O*: 0.278, *Na*: 0.001, *S*: 0.001, *Cl*: 0.001	0.024	30	0.1
2.	Blood (whole)	*H*: 0.102, *C*: 0.110, *N*: 0.033, *O*: 0.745, *Na*: 0.001, *P*: 0.001, *S*: 0.002, *Cl*: 0.003, *K*: 0.002, *Fe*: 0.001	0.029	26	0.1
3.	Cortical bone	*H*: 0.034, *C*: 0.155, *N*: 0.042, *O*: 0.435, *Na*: 0.001, *Mg*: 0.002, *P*: 0.103, *S*: 0.003, *Ca*: 0.225	0.055	18	0.2
4.	Brain (grey/ white matter)	*H*: 0.107, *C*: 0.145, *N*: 0.022, *O*: 0.712, *Na*: 0.002, *P*: 0.004, *S*: 0.002, *Cl*: 0.003, *K*: 0.003	0.028	26	0.1
5.	Breast tissue	*H*: 0.106, *C*: 0.332, *N*: 0.030, *O*: 0.527, *Na*: 0.001, *P*: 0.001, *S*: 0.002, *Cl*: 0.001	0.026	28	0.1
6.	Eye lens	*H*: 0.096, *C*: 0.195, *N*: 0.057, *O*: 0.646, *Na*: 0.001, *P*: 0.001, *S*: 0.003, *Cl*: 0.001	0.027	26	0.1
7.	Lung tissue	*H*: 0.103, *C*: 0.105, *N*: 0.031, *O*: 0.749, *Na*: 0.002, *P*: 0.002, *S*: 0.003, *Cl*: 0.003, *K*: 0.002	0.029	26	0.1
8.	Skeletal muscle	*H*: 0.102, *C*: 0.143, *N*: 0.034, *O*: 0.710, *Na*: 0.001, *P*: 0.002, *S*: 0.003, *Cl*: 0.001, *K*: 0.004	0.028	26	0.1
9.	Ovary	*H*: 0.105, *C*: 0.093, *N*: 0.024, *O*: 0.768, *Na*: 0.002, *P*: 0.002, *S*: 0.002, *Cl*: 0.002, *K*: 0.002	0.028	26	0.1
10.	Testis	*H*: 0.106, *C*: 0.099, *N*: 0.020, *O*: 0.766, *Na*: 0.002, *P*: 0.001, *S*: 0.002, *Cl*: 0.002, *K*: 0.002	0.028	26	0.1
11.	Soft tissue	*H*: 0.102, *C*: 0.143, *N*: 0.034, *O*: 0.708, *Na*: 0.002, *P*: 0.003, *S*: 0.003, *Cl*: 0.002, *K*: 0.003	0.028	26	0.1
12.	Soft tissue (4‐component)	*H*: 0.101174, *C*: 0.111, *N*: 0.026, *O*: 0.761826	0.027	26	0.1

### A.1 Computation of the equivalent atomic number, $Z_{\text{eq}}$

The equivalent atomic number, $Z_{\text{eq}}$, is a parameter assigned to a compound or mixture by giving heavy weight to Compton scattering, since the buildup factor is a consequence of multiple scattering for which the main contribution is due to Compton scattering. The value of $Z_{\text{eq}}$ for a given material is energy dependent.

Values of the Compton partial mass attenuation coefficient, ${\left(\mu /\rho \right)}_{\text{Comp}}$, and the total mass attenuation coefficient, ${\left(\mu /\rho \right)}_{\text{total}}$, have been obtained for the elements Z=4−30, and for the human organs and tissues using the WinXCom program.^(^ ^6^ ^,^ ^7^ ^)^ The equivalent atomic number, $Z_{\text{eq}}$, for a given human organ or tissue is then calculated by matching the ratio, ${\left(\mu /\rho \right)}_{\text{Comp}}/{\left(\mu /\rho \right)}_{\text{total}}$, of that human organ or tissue at a given energy with the corresponding ratio of a pure element at the same energy. If this ratio lies between the two ratios for known elements, then the value of $Z_{\text{eq}}$ is interpolated using the following formula:^(^ ^12^ ^,^ ^13^ ^)^

(1) $$Z_{\text{eq}}=\frac{Z_{1}(\log R_{2}-\log R)+Z_{2}(\log R-\log R_{1})}{\log R_{2}-\log R_{1}}$$

where $Z_{1}$ and $Z_{2}$ are the atomic numbers of two elements, $R_{1}$ and $R_{2}$ are the ratios ${\left(\mu /\rho \right)}_{\text{Comp}}/{\left(\mu /\rho \right)}_{\text{total}}$ for these elements, and *R* is the corresponding ratio for a given human organ or tissue at a given energy, which lies between $R_{1}$ and $R_{2}$ (nearest neighbours of R).

Example: Consider cortical bone and incident photons of energy 0.03 MeV. The ratio ${\left(\mu /\rho \right)}_{\text{Comp}}/{\left(\mu /\rho \right)}_{\text{total}}$ for cortical bone is R=0.1313, which should be compared with $R_{1}=0.1438$ and $R_{2}=0.1145$ for the elements $Z_{1}=13$ and $Z_{2}=14$. It follows from Eq. (1) that $Z_{\text{eq}}=13.40$.

Values of the equivalent atomic number, $Z_{\text{eq}}$, obtained in this way for human organs and tissues are given in Table 2.

**Table 2:** Equivalent atomic numbers, $Z_{\text{eq}}$, of selected human organs and tissues. Sample numbers as for Table 1.

*Energy (MeV)*	*1*	*2*	*3*	*4*	*5*	*6*	*7*	*8*	*9*	*10*	*11*	*12*
0.015	6.39	7.56	12.99	7.50	6.96	7.25	7.52	7.49	7.50	7.47	7.50	7.28
0.020	6.41	7.61	13.18	7.54	7.00	7.28	7.56	7.53	7.53	7.51	7.54	7.31
0.030	6.43	7.66	13.40	7.58	7.05	7.31	7.60	7.57	7.57	7.54	7.58	7.33
0.040	6.44	7.68	13.53	7.61	7.06	7.32	7.62	7.59	7.59	7.56	7.60	7.33
0.050	6.45	7.71	13.62	7.62	7.08	7.33	7.64	7.61	7.60	7.57	7.61	7.34
0.060	6.46	7.72	13.69	7.64	7.08	7.34	7.65	7.62	7.61	7.58	7.63	7.34
0.080	6.47	7.75	13.78	7.66	7.09	7.34	7.66	7.64	7.62	7.59	7.65	7.34
0.100	6.48	7.77	13.84	7.67	7.10	7.35	7.68	7.65	7.63	7.60	7.66	7.35
0.150	6.49	7.80	13.94	7.69	7.11	7.36	7.70	7.67	7.65	7.62	7.68	7.35
0.200	6.49	7.81	14.00	7.71	7.12	7.37	7.71	7.69	7.66	7.63	7.69	7.35
0.300	6.50	7.83	14.06	7.72	7.12	7.37	7.72	7.70	7.67	7.64	7.71	7.36
0.400	6.50	7.84	14.09	7.73	7.13	7.38	7.73	7.71	7.68	7.64	7.71	7.36
0.500	6.50	7.85	14.10	7.73	7.13	7.38	7.73	7.71	7.68	7.65	7.72	7.36
0.600	6.51	7.86	14.11	7.74	7.13	7.38	7.73	7.71	7.68	7.65	7.72	7.36
0.800	6.51	7.86	14.12	7.74	7.13	7.38	7.74	7.72	7.68	7.65	7.72	7.36
1.000	6.51	7.86	14.12	7.74	7.13	7.38	7.74	7.72	7.68	7.65	7.72	7.36
1.500	5.56	6.65	11.43	6.55	6.13	6.47	6.64	6.58	6.63	6.60	6.59	6.55
2.000	5.53	6.59	10.78	6.49	6.09	6.43	6.59	6.53	6.58	6.55	6.53	6.51
3.000	5.52	6.57	10.61	6.48	6.08	6.42	6.57	6.52	6.57	6.54	6.52	6.51
4.000	5.51	6.57	10.56	6.47	6.07	6.42	6.57	6.51	6.56	6.53	6.52	6.50
5.000	5.51	6.57	10.53	6.47	6.07	6.41	6.57	6.51	6.56	6.53	6.51	6.50
6.000	5.51	6.56	10.52	6.47	6.07	6.41	6.56	6.50	6.55	6.53	6.51	6.50
8.000	5.51	6.56	10.51	6.46	6.06	6.41	6.56	6.50	6.55	6.53	6.51	6.49
10.000	5.51	6.56	10.50	6.46	6.06	6.41	6.56	6.50	6.55	6.52	6.50	6.49
15.000	5.51	6.55	10.49	6.46	6.06	6.40	6.55	6.49	6.54	6.52	6.50	6.49

### A.2 Computation of the geometric progression (GP) fitting parameters

The $Z_{\text{eq}}$ values obtained in Section A.1 above were used to calculate GP fitting parameters (*a, b, c, d, and* $X_{k}$) for energy absorption buildup factors using the following interpolation formula:^(^ ^12^ ^,^ ^13^ ^)^

(2) $$P=\frac{P_{1}(\log Z_{2}-\log Z_{\text{eq}})+P_{2}(\log Z_{\text{eq}}-\log Z_{1})}{\log Z_{2}-\log Z_{1}}$$

where $Z_{1}$ and $Z_{2}$ are the atomic numbers of elements between which the equivalent atomic number, $Z_{\text{eq}}$, of a given human organ or tissue lies, and $P_{1}$ and $P_{2}$ are the values of GP fitting parameters corresponding to the atomic numbers $Z_{1}$ and $Z_{2}$, respectively, at a given energy.

GP fitting parameters for the pure elements were taken from the standard reference ANSI/ANS‐6.4.3‐1991.^(^ ^2^ ^)^ The resulting energy absorption GP fitting parameters for human organs and tissues are given in Tables 3–8.

**Table 3:** Energy absorption GP fitting parameters for adipose tissue and blood (whole) in the energy range 0.015 MeV–15 MeV.

*Energy*		*Adipose Tissue*					*Blood (whole)*			
*(MeV)*	*a*	*b*	*c*	*d*	$X_{k}$	*a*	*b*	*c*	*d*	$X_{k}$
0.015	0.157	1.332	0.509	−0.0780	14.48	0.183	1.189	0.450	−0.0929	13.75
0.020	0.101	1.752	0.669	−0.0492	15.78	0.153	1.436	0.531	−0.0776	14.63
0.030	0.009	3.267	1.013	−0.0122	14.14	0.095	2.350	0.718	−0.0436	13.02
0.040	−0.092	4.567	1.521	0.0376	13.96	−0.007	3.450	1.075	−0.0042	13.40
0.050	−0.139	5.265	1.861	0.0589	14.24	−0.073	4.372	1.402	0.0280	13.56
0.060	−0.169	5.290	2.111	0.0742	14.24	−0.116	4.846	1.667	0.0503	13.70
0.080	−0.195	4.887	2.360	0.0828	14.14	−0.159	4.936	1.987	0.0710	13.64
0.100	−0.196	4.481	2.400	0.0802	14.68	−0.173	4.635	2.119	0.0770	13.88
0.150	−0.197	3.637	2.394	0.0780	14.68	−0.174	3.888	2.153	0.0705	14.40
0.200	−0.187	3.271	2.278	0.0759	14.79	−0.175	3.365	2.138	0.0722	14.13
0.300	−0.171	2.820	2.091	0.0681	14.57	−0.160	2.892	1.987	0.0630	14.12
0.400	−0.152	2.624	1.911	0.0616	14.51	−0.145	2.646	1.854	0.0574	14.11
0.500	−0.138	2.459	1.793	0.0585	15.04	−0.133	2.477	1.751	0.0530	14.26
0.600	−0.122	2.380	1.672	0.0479	14.59	−0.121	2.366	1.659	0.0479	14.30
0.800	−0.107	2.200	1.553	0.0447	14.15	−0.101	2.212	1.522	0.0401	14.36
1.000	−0.089	2.098	1.444	0.0379	14.47	−0.086	2.105	1.427	0.0348	14.27
1.500	−0.061	1.938	1.279	0.0271	14.32	−0.060	1.934	1.276	0.0263	14.35
2.000	−0.039	1.838	1.174	0.0165	14.35	−0.039	1.838	1.172	0.0161	14.18
3.000	−0.012	1.715	1.052	0.0039	13.78	−0.012	1.711	1.054	0.0036	13.32
4.000	0.003	1.628	0.988	−0.0027	13.81	0.005	1.628	0.984	−0.0049	13.70
5.000	0.015	1.567	0.943	−0.0083	14.48	0.018	1.566	0.938	−0.0115	14.15
6.000	0.029	1.522	0.901	−0.0166	12.73	0.023	1.505	0.920	−0.0169	15.14
8.000	0.038	1.440	0.871	−0.0192	11.66	0.037	1.430	0.875	−0.0204	12.07
10.000	0.040	1.383	0.858	−0.0215	14.41	0.039	1.370	0.865	−0.0222	14.33
15.000	0.047	1.289	0.836	−0.0296	15.04	0.048	1.277	0.839	−0.0342	15.39

**Table 4:** Energy absorption GP fitting parameters for cortical bone and brain (grey/white matter) in the energy range 0.015 MeV–15 MeV.

*Energy*		*Cortical Bone*					*Brain (grey/white matter)*			
*(MeV)*	*a*	*b*	*c*	*d*	$X_{k}$	*a*	*b*	*c*	*d*	$X_{k}$
0.015	0.240	1.029	0.365	−0.1699	14.11	0.181	1.194	0.454	−0.0913	13.84
0.020	0.229	1.068	0.364	−0.1242	14.89	0.151	1.449	0.536	−0.0764	14.66
0.030	0.210	1.207	0.407	−0.1147	14.20	0.090	2.388	0.731	−0.0429	13.34
0.040	0.181	1.448	0.472	−0.1003	14.71	−0.012	3.507	1.096	−0.0019	13.44
0.050	0.130	1.772	0.591	−0.0709	16.03	−0.077	4.430	1.427	0.0301	13.61
0.060	0.130	2.256	0.628	−0.0781	13.92	−0.120	4.880	1.696	0.0524	13.70
0.080	0.057	3.131	0.843	−0.0454	14.02	−0.162	4.933	2.015	0.0723	13.62
0.100	0.005	3.754	1.045	−0.0235	13.73	−0.174	4.638	2.134	0.0770	13.95
0.150	−0.061	4.048	1.353	0.0105	13.96	−0.176	3.871	2.171	0.0712	14.40
0.200	−0.087	3.678	1.506	0.0242	14.37	−0.175	3.365	2.145	0.0727	14.21
0.300	−0.099	3.111	1.579	0.0287	14.43	−0.161	2.885	1.996	0.0635	14.13
0.400	−0.099	2.783	1.567	0.0295	14.63	−0.146	2.642	1.859	0.0577	14.12
0.500	−0.096	2.572	1.537	0.0296	14.92	−0.134	2.475	1.756	0.0533	14.24
0.600	−0.091	2.434	1.494	0.0280	15.00	−0.121	2.366	1.662	0.0482	14.29
0.800	−0.081	2.242	1.422	0.0264	15.10	−0.101	2.211	1.525	0.0405	14.34
1.000	−0.071	2.123	1.358	0.0243	14.98	−0.086	2.105	1.427	0.0349	14.33
1.500	−0.054	1.938	1.251	0.0204	14.38	−0.060	1.935	1.276	0.0263	14.34
2.000	−0.034	1.845	1.155	0.0126	14.74	−0.039	1.839	1.172	0.0158	14.23
3.000	−0.009	1.706	1.050	−0.0009	11.05	−0.012	1.712	1.053	0.0034	13.51
4.000	0.009	1.615	0.983	−0.0114	13.14	0.005	1.627	0.985	−0.0045	13.56
5.000	0.019	1.544	0.945	−0.0147	12.75	0.017	1.565	0.939	−0.0107	14.29
6.000	0.025	1.483	0.926	−0.0269	15.85	0.024	1.507	0.917	−0.0171	14.69
8.000	0.033	1.391	0.902	−0.0223	12.29	0.036	1.430	0.877	−0.0196	12.08
10.000	0.037	1.329	0.890	−0.0284	13.93	0.039	1.371	0.864	−0.0222	14.32
15.000	0.039	1.230	0.885	−0.0333	14.72	0.048	1.278	0.838	−0.0339	15.48

**Table 5:** Energy absorption GP fitting parameters for breast tissue and eye lens in the energy range 0.015 MeV–15 MeV.

*Energy*		*Breast Tissue*					*Eye Lens*			
*(MeV)*	*a*	*b*	*c*	*d*	$X_{k}$	*a*	*b*	*c*	*d*	$X_{k}$
0.015	0.163	1.241	0.486	−0.0784	14.56	0.172	1.214	0.469	−0.0851	14.19
0.020	0.134	1.558	0.578	−0.0662	14.90	0.143	1.500	0.555	−0.0716	14.77
0.030	0.055	2.686	0.834	−0.0378	15.83	0.073	2.537	0.782	−0.0404	14.58
0.040	−0.047	3.931	1.252	0.0149	13.76	−0.030	3.726	1.177	0.0068	13.61
0.050	−0.107	4.835	1.604	0.0451	13.92	−0.093	4.644	1.521	0.0380	13.77
0.060	−0.147	5.108	1.889	0.0666	13.76	−0.134	5.002	1.799	0.0600	13.73
0.080	−0.182	4.916	2.194	0.0809	13.46	−0.173	4.924	2.112	0.0770	13.53
0.100	−0.182	4.657	2.230	0.0770	14.40	−0.178	4.649	2.187	0.0770	14.20
0.150	−0.187	3.770	2.277	0.0755	14.41	−0.182	3.814	2.231	0.0736	14.41
0.200	−0.178	3.365	2.186	0.0755	14.70	−0.177	3.365	2.169	0.0743	14.49
0.300	−0.167	2.849	2.045	0.0662	14.20	−0.164	2.864	2.024	0.0650	14.17
0.400	−0.150	2.624	1.891	0.0598	14.19	−0.148	2.632	1.878	0.0589	14.16
0.500	−0.137	2.464	1.780	0.0551	14.14	−0.136	2.469	1.770	0.0544	14.18
0.600	−0.123	2.364	1.677	0.0496	14.25	−0.122	2.365	1.670	0.0490	14.27
0.800	−0.104	2.204	1.541	0.0429	14.23	−0.103	2.207	1.534	0.0419	14.27
1.000	−0.086	2.107	1.429	0.0354	14.65	−0.086	2.106	1.428	0.0352	14.51
1.500	−0.060	1.940	1.275	0.0265	14.30	−0.060	1.936	1.276	0.0264	14.33
2.000	−0.037	1.842	1.168	0.0146	14.47	−0.038	1.839	1.171	0.0156	14.27
3.000	−0.011	1.714	1.051	0.0028	14.35	−0.012	1.712	1.053	0.0034	13.63
4.000	0.003	1.626	0.989	−0.0024	12.94	0.005	1.627	0.986	−0.0042	13.47
5.000	0.015	1.564	0.946	−0.0074	14.93	0.017	1.565	0.940	−0.0103	14.38
6.000	0.029	1.517	0.903	−0.0184	12.70	0.025	1.509	0.915	−0.0173	14.41
8.000	0.033	1.430	0.883	−0.0163	12.11	0.036	1.430	0.878	−0.0192	12.08
10.000	0.040	1.377	0.860	−0.0221	14.32	0.039	1.372	0.864	−0.0222	14.32
15.000	0.047	1.282	0.838	−0.0326	15.85	0.048	1.278	0.838	−0.0337	15.53

**Table 6:** Energy absorption GP fitting parameters for lung tissue and skeletal muscle in the energy range 0.015 MeV–15 MeV.

*Energy*		*Lung Tissue*					*Skeletal Muscle*			
*(MeV)*	*a*	*b*	*c*	*d*	$X_{k}$	*a*	*b*	*c*	*d*	$X_{k}$
0.015	0.182	1.192	0.453	−0.0920	13.80	0.181	1.195	0.455	−0.0911	13.86
0.020	0.151	1.444	0.534	−0.0768	14.65	0.150	1.451	0.537	−0.0761	14.67
0.030	0.092	2.379	0.728	−0.0431	13.26	0.089	2.397	0.734	−0.0428	13.41
0.040	−0.011	3.496	1.092	−0.0023	13.44	−0.013	3.520	1.101	−0.0014	13.45
0.050	−0.076	4.421	1.424	0.0298	13.60	−0.078	4.443	1.433	0.0306	13.62
0.060	−0.119	4.876	1.692	0.0521	13.70	−0.121	4.887	1.702	0.0529	13.71
0.080	−0.162	4.933	2.012	0.0722	13.62	−0.162	4.933	2.021	0.0726	13.61
0.100	−0.174	4.638	2.133	0.0770	13.95	−0.174	4.639	2.137	0.0770	13.97
0.150	−0.176	3.871	2.170	0.0712	14.40	−0.176	3.867	2.175	0.0714	14.40
0.200	−0.175	3.365	2.145	0.0727	14.21	−0.176	3.365	2.147	0.0728	14.23
0.300	−0.161	2.885	1.996	0.0635	14.13	−0.161	2.884	1.997	0.0636	14.13
0.400	−0.146	2.642	1.860	0.0578	14.12	−0.146	2.642	1.861	0.0578	14.12
0.500	−0.134	2.475	1.756	0.0533	14.24	−0.134	2.474	1.757	0.0534	14.24
0.600	−0.121	2.366	1.662	0.0482	14.29	−0.121	2.366	1.663	0.0482	14.29
0.800	−0.101	2.211	1.525	0.0406	14.33	−0.101	2.211	1.526	0.0406	14.33
1.000	−0.086	2.105	1.428	0.0349	14.33	−0.086	2.105	1.428	0.0349	14.34
1.500	−0.060	1.934	1.276	0.0263	14.35	−0.060	1.935	1.276	0.0263	14.34
2.000	−0.039	1.838	1.172	0.0161	14.18	−0.039	1.839	1.172	0.0159	14.21
3.000	−0.012	1.711	1.054	0.0036	13.32	−0.012	1.711	1.054	0.0035	13.44
4.000	0.005	1.628	0.984	−0.0049	13.70	0.005	1.628	0.985	−0.0046	13.61
5.000	0.018	1.566	0.938	−0.0115	14.15	0.018	1.566	0.939	−0.0110	14.24
6.000	0.023	1.505	0.920	−0.0169	15.14	0.024	1.506	0.918	−0.0170	14.86
8.000	0.037	1.430	0.875	−0.0204	12.07	0.037	1.430	0.876	−0.0199	12.07
10.000	0.039	1.370	0.865	−0.0222	14.33	0.039	1.371	0.865	−0.0222	14.33
15.000	0.048	1.277	0.839	−0.0342	15.39	0.048	1.277	0.839	−0.0340	15.44

**Table 7:** Energy absorption GP fitting parameters for ovary and testis in the energy range 0.015 MeV–15 MeV.

*Energy*		*Ovary*					*Testis*			
*(MeV)*	*a*	*b*	*c*	*d*	$X_{k}$	*a*	*b*	*c*	*d*	$X_{k}$
0.015	0.181	1.194	0.454	−0.0913	13.84	0.180	1.196	0.456	−0.0907	13.88
0.020	0.151	1.450	0.536	−0.0762	14.66	0.150	1.455	0.538	−0.0757	14.67
0.030	0.089	2.397	0.734	−0.0428	13.41	0.088	2.411	0.739	−0.0425	13.54
0.040	−0.013	3.524	1.102	−0.0012	13.46	−0.015	3.545	1.110	−0.0004	13.47
0.050	−0.079	4.449	1.436	0.0308	13.62	−0.080	4.469	1.445	0.0316	13.64
0.060	−0.121	4.892	1.706	0.0531	13.71	−0.122	4.903	1.716	0.0539	13.71
0.080	−0.163	4.932	2.025	0.0728	13.61	−0.164	4.931	2.035	0.0733	13.60
0.100	−0.175	4.640	2.140	0.0770	13.98	−0.175	4.641	2.145	0.0770	14.00
0.150	−0.176	3.863	2.179	0.0715	14.40	−0.177	3.858	2.184	0.0718	14.40
0.200	−0.176	3.365	2.149	0.0729	14.25	−0.176	3.365	2.151	0.0731	14.27
0.300	−0.161	2.882	2.000	0.0637	14.13	−0.161	2.880	2.002	0.0639	14.14
0.400	−0.146	2.641	1.862	0.0579	14.13	−0.146	2.640	1.864	0.0580	14.13
0.500	−0.134	2.474	1.758	0.0535	14.23	−0.134	2.473	1.759	0.0536	14.23
0.600	−0.121	2.366	1.663	0.0483	14.29	−0.121	2.366	1.664	0.0484	14.29
0.800	−0.102	2.210	1.527	0.0407	14.33	−0.102	2.210	1.527	0.0409	14.32
1.000	−0.086	2.105	1.428	0.0349	14.36	−0.086	2.105	1.428	0.0350	14.37
1.500	−0.060	1.935	1.276	0.0263	14.35	−0.060	1.935	1.276	0.0263	14.35
2.000	−0.039	1.838	1.172	0.0160	14.18	−0.039	1.838	1.172	0.0160	14.20
3.000	−0.012	1.711	1.054	0.0036	13.34	−0.012	1.711	1.054	0.0035	13.39
4.000	0.005	1.628	0.984	−0.0049	13.69	0.005	1.628	0.984	−0.0048	13.65
5.000	0.018	1.566	0.938	−0.0114	14.16	0.018	1.566	0.938	−0.0112	14.20
6.000	0.023	1.505	0.920	−0.0169	15.10	0.023	1.506	0.919	−0.0170	14.98
8.000	0.037	1.430	0.875	−0.0203	12.07	0.037	1.430	0.876	−0.0201	12.07
10.000	0.039	1.370	0.865	−0.0222	14.33	0.039	1.370	0.865	−0.0222	14.33
15.000	0.048	1.277	0.839	−0.0341	15.40	0.048	1.277	0.839	−0.0341	15.42

**Table 8:** Energy absorption GP fitting parameters for soft tissue and soft tissue (4‐component) in the energy range 0.015 MeV–15 MeV.

*Energy*		*Soft Tissue*					*Soft Tissue (4‐component)*			
*(MeV)*	*a*	*b*	*c*	*d*	$X_{k}$	*a*	*b*	*c*	*d*	$X_{k}$
0.015	0.181	1.194	0.454	−0.0913	13.84	0.173	1.212	0.467	−0.0858	14.15
0.020	0.151	1.449	0.536	−0.0763	14.66	0.144	1.495	0.553	−0.0721	14.76
0.030	0.090	2.392	0.733	−0.0429	13.37	0.074	2.528	0.779	−0.0405	14.51
0.040	−0.012	3.514	1.099	−0.0016	13.45	−0.029	3.718	1.174	0.0065	13.60
0.050	−0.078	4.437	1.430	0.0304	13.61	−0.092	4.640	1.519	0.0379	13.77
0.060	−0.120	4.884	1.699	0.0527	13.71	−0.134	5.001	1.798	0.0600	13.73
0.080	−0.162	4.933	2.018	0.0725	13.61	−0.173	4.924	2.113	0.0770	13.53
0.100	−0.174	4.639	2.136	0.0770	13.96	−0.178	4.649	2.188	0.0770	14.20
0.150	−0.176	3.869	2.173	0.0713	14.40	−0.182	3.812	2.232	0.0737	14.41
0.200	−0.175	3.365	2.146	0.0728	14.22	−0.177	3.365	2.170	0.0744	14.50
0.300	−0.161	2.884	1.997	0.0636	14.13	−0.164	2.863	2.025	0.0651	14.17
0.400	−0.146	2.642	1.860	0.0578	14.12	−0.148	2.631	1.879	0.0590	14.17
0.500	−0.134	2.474	1.756	0.0534	14.24	−0.136	2.468	1.770	0.0544	14.18
0.600	−0.121	2.366	1.662	0.0482	14.29	−0.122	2.365	1.671	0.0490	14.27
0.800	−0.101	2.211	1.526	0.0406	14.33	−0.103	2.207	1.535	0.0420	14.27
1.000	−0.086	2.105	1.428	0.0349	14.34	−0.086	2.106	1.428	0.0352	14.52
1.500	−0.060	1.935	1.276	0.0263	14.34	−0.060	1.935	1.276	0.0263	14.34
2.000	−0.039	1.839	1.172	0.0159	14.21	−0.039	1.839	1.172	0.0158	14.22
3.000	−0.012	1.711	1.054	0.0035	13.43	−0.012	1.711	1.054	0.0035	13.46
4.000	0.005	1.628	0.985	−0.0047	13.62	0.005	1.628	0.985	−0.0046	13.60
5.000	0.018	1.566	0.938	−0.0111	14.23	0.018	1.566	0.939	−0.0109	14.25
6.000	0.024	1.506	0.919	−0.0170	14.89	0.024	1.507	0.918	−0.0171	14.82
8.000	0.037	1.430	0.876	−0.0200	12.07	0.037	1.430	0.876	−0.0198	12.07
10.000	0.039	1.371	0.865	−0.0222	14.33	0.039	1.371	0.865	−0.0222	14.33
15.000	0.048	1.277	0.839	−0.0340	15.44	0.048	1.277	0.839	−0.0340	15.45

### A.3 Computation of the energy absorption buildup factor

Finally, the energy absorption buildup factor, B, was calculated by using the following GP fitting formula given by Harima et al.:^(^ ^9^ ^)^

(3) $$B(E,x)=\{\begin{array}{ll} 1+\frac{\left(b-1)(K^{x}-1\right)}{K-1} & \text{for}\;K\neq 1\\ 1+(b-1)x & \text{for}\;K=1 \end{array}$$

(4) $$\text{where}\;\;\;K(E,x)=c\;x^{a}+d\frac{\tanh(x/X_{K}-2)-\tanh(-2)}{1-\tanh(-2)}\;\;\;\text{for}\;\;x\leq 40\;\text{mfp}$$

where *E* is the source energy, *x* is the penetration depth in units of mfp (mean free path), and *a, b, c, d and* $X_{k}$ are GP fitting parameters. The value of the parameter *b* corresponds to the buildup factor at 1 mfp. The variation of the parameter *K* with penetration depth represents the photon dose multiplication and change in the shape of the spectrum.

## III. RESULtS & DISCUSSION

### A. Buildup factors of water

In order to check the reliability of our method, we have calculated energy absorption buildup factors of water. For this compound, tabulated values of energy absorption buildup factors are available in the ANSI/ANS‐6.4.3 standard.^(^ ^2^ ^)^

**Figure 1:**
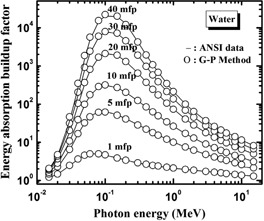
Energy absorption buildup factors of water obtained in the present work (circles) compared with those of the ANSI/ANS‐6.4.3 standard (solid line) at selected penetration depths (in units of mfp).

Figure 1 shows the energy dependence of the energy absorption buildup factor for water, as given by the present method and by the ANSI/ANS‐6.4.3 standard for some selected penetration depths from 1 to 40 mfp. It is seen that there is a good agreement between the ANSI/ANS‐6.4.3 standard data and present results.

In Table 9, we compare our calculated energy absorption buildup factors for water with those of ANSI/ANS‐6.4.3 at energies 0.015–15 MeV. The absolute percentage difference is varying between 0.07% and 4.29%. Hence, we conclude that the energy absorption buildup factors generated by our computational procedure are in good agreement, except for occasional slight differences, with those given by ANSI/ANS‐6.4.3 standard data for water.

**Table 9:** Energy absorption buildup factors of the water obtained by the present work compared with those of the ANSI/ANS‐6.4.3 standard.^(^ ^2^ ^)^


	Energy = 0.15 MeV			Energy=0.3 Mev			Energy=0.5 Mev		
*x (mfp)*	*ANSI*	*GP method*	*% error*	*ANSI*	*GP method*	*% error*	*ANSI*	*GP method*	*% error*
1	3.91	3.84	1.84	2.84	2.87	1.14	2.45	2.47	0.85
2	9.36	9.37	0.07	6.25	6.24	0.16	4.87	4.84	0.71
3	18.60	18.30	1.62	11.50	11.34	1.43	8.29	8.12	2.08
4	32.50	31.60	2.76	19.00	18.49	2.66	12.70	12.40	2.38
5	52.00	50.39	3.09	28.80	28.07	2.54	18.10	17.76	1.90
6	77.90	75.87	2.60	41.20	40.41	1.91	24.60	24.26	1.37
7	111.00	109.33	1.50	56.50	55.86	1.13	32.20	31.97	0.73
8	153.00	152.10	0.59	75.00	74.73	0.36	40.80	40.91	0.26
10	268.00	270.97	1.11	122.00	123.82	1.49	61.80	62.57	1.25
15	805.00	825.45	2.54	318.00	322.26	1.34	137.00	138.40	1.02
20	1890.00	1897.57	0.40	656.00	647.46	1.30	247.00	244.31	1.09
25	3840.00	3804.69	0.92	1180.00	1148.35	2.68	395.00	386.69	2.10
30	7050.00	7158.55	1.54	1930.00	1916.63	0.69	582.00	579.99	0.34
35	12100.00	12619.08	4.29	2950.00	2992.81	1.45	809.00	822.03	1.61
40	19600.00	19968.27	1.88	4280.00	4205.56	1.74	1080.00	1067.38	1.17

	Energy=1 Mev			Energy=5 Mev			Energy=15 Mev		
*x (mfp)*	*ANSI*	*G‐P method*	*% error*	*ANSI*	*GP method*	*% error*	*ANSI*	*GP method*	*% error*
1	2.08	2.11	1.24	1.57	1.57	0.26	1.29	1.28	1.06
2	3.62	3.59	0.72	2.10	2.10	0.14	1.51	1.52	0.39
3	5.50	5.41	1.62	2.62	2.62	0.15	1.72	1.74	0.96
4	7.66	7.54	1.56	3.12	3.13	0.47	1.93	1.94	0.76
5	10.10	9.97	1.28	3.63	3.64	0.21	2.14	2.14	0.21
6	12.80	12.69	0.87	4.14	4.14	0.10	2.34	2.34	0.07
7	15.70	15.68	0.11	4.64	4.63	0.21	2.53	2.53	0.08
8	18.90	18.94	0.19	5.14	5.12	0.34	2.73	2.72	0.55
10	26.00	26.16	0.61	6.14	6.10	0.58	3.11	3.08	0.81
15	47.40	47.62	0.47	8.62	8.57	0.58	4.04	4.01	0.79
20	73.50	72.87	0.85	11.10	11.06	0.39	4.93	4.94	0.24
25	104.00	102.17	1.76	13.50	13.52	0.13	5.81	5.85	0.62
30	138.00	136.84	0.84	15.90	15.91	0.04	6.64	6.66	0.32
35	175.00	175.87	0.50	18.30	18.27	0.18	7.42	7.39	0.46
40	214.00	213.56	0.21	20.70	20.73	0.13	8.09	8.11	0.21

### B. Buildup factors of human organs and tissues

The computed energy absorption GP fitting parameters (Tables 3–8) were used to generate energy absorption buildup factors. In the following paragraphs, we discuss how the buildup factors vary with incident photon energy, chemical composition, and effective atomic number.

#### B.1 Energy absorption buildup factor as a function of incident photon energy

**Figure 2:**
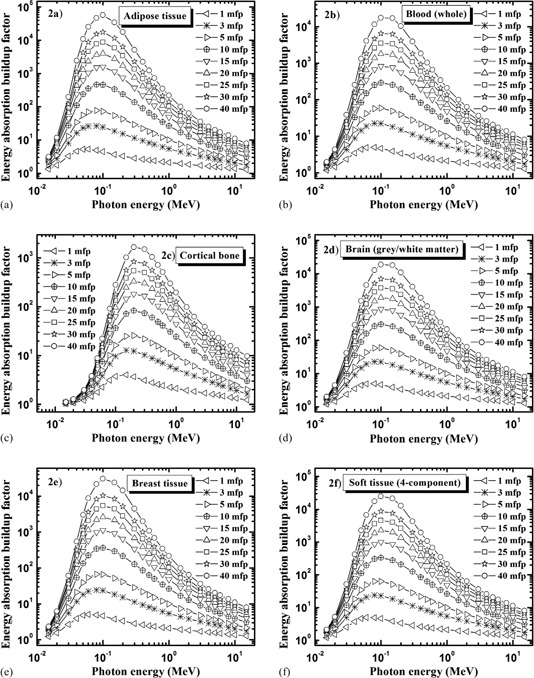
Variation of the energy absorption buildup factor with photon energy for some selected penetration depths up to 40 mfp: (a) adipose tissue, (b) blood (whole), (c) cortical bone, (d) brain (grey/white matter), (e) breast tissue, (f) soft tissue (4‐component).

Figures 2(a)–2(f) show the energy dependence of energy absorption buildup factor at some selected penetration depths from 1–40 mfp for human organs and tissues (Table 1). Brain (grey/white matter), lung tissue, skeletal muscle, ovary, testis, and soft tissue are seen to constitute a group of materials having similar energy dependence, and the same is true for breast tissue and the eye lens. This is because each of these two sets consists of materials having similar chemical composition (see Table 1). Therefore, graphs are shown only for adipose tissue, blood (whole), cortical bone, brain (grey/white matter), breast tissue, and soft tissue (4‐component).

The buildup factor is small for all penetration depths at low energies, $E\;\leq E_{\text{pe}}$, where $E_{\text{pe}}$ is the energy for which the interaction cross sections for photoelectric absorption and Compton scattering are equal. Photoelectric absorption is the dominating photon interaction process at low energies, resulting in a fast removal of the incident low‐energy photons and thus not allowing any appreciable buildup of photons. In Table 1, we give values of $E_{\text{pe}}$ derived from the WinXCom program.^(^ ^6^ ^,^ ^7^ ^)^

The buildup factor reaches large values at medium energies, $E_{\text{pe}}\;<E\;<E_{\text{pp}}$, where $E_{\text{pp}}$ is the energy for which the interaction cross sections for Compton scattering and pair production are equal. At these energies (i.e. in the energy range 0.04–1 MeV), the buildup factor reaches large values for a given penetration depth. This is due to the fact that Compton scattering is known to give rise to extensive multiple scattering of photons, which just degrades the photon energy and fails to remove a photon completely. Because of this, the multiple scattered photons exist for a longer time in the material, which leads to a higher value of buildup factor. This also explains the broad peak with a maximum at energy $E_{\text{peak}}$ in the range 0.05−0.2MeV. The value of $E_{\text{peak}}$ in each case is given in Table 1. Figures 2(a)–(f) also show that the buildup factor of adipose tissue, brain (grey/white matter), blood (whole), breast tissue, and soft tissue (4‐component) reaches very high values, on the order of $10^{4}-10^{5}$, for the largest penetration depth (40 mfp). The high value is due to the fact that these organs/tissues are low‐Z materials, characterized by strong Compton scattering.

Similar to photoelectric absorption, the buildup factor is small at high energies $E>E_{\text{pp}}$ where pair production is dominating, resulting in strong absorption of photons. Values of the limiting energy $E_{\text{pp}}$ are given in Table 1, which are obtained from WinXCom program.^(^ ^6^ ^,^ ^7^ ^)^

Sidhu et al.^(^ ^14^ ^)^ have applied the GP fitting method in the past for muscle, tissue, and bone (compact) with some slightly different chemical composition.^(^ ^15^ ^)^ In our study, we have taken the chemical composition data for skeletal muscle, soft tissue, soft tissue (4‐component), and cortical bone from the standard references.^(^ ^10^ ^,^ ^11^ ^)^ However, though the names of human organ and tissues referenced by Sidhu et al. sound similar, they have quite different compositions (e.g., cortical bone is different from bone (compact), etc.). Hence, there are discrepancies in the computed values of GP fitting parameters and buildup factors of corresponding human organ and tissues.

#### B.2 Effect of the chemical composition

**Figure 3:**
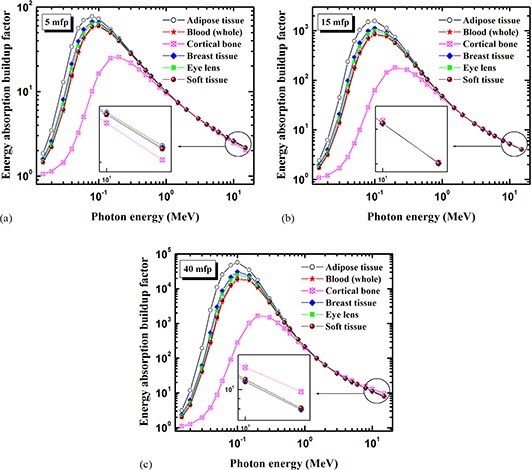
Comparison of energy absorption buildup factors for human organs and tissues at: (a) 5 mfp, (b) 15 mfp, and (c) 40 mfp.

Figures 3(a)–3(c) show the energy absorption buildup factors of human organs and tissues compared at the penetration depths 5, 15, and 40 mfp.

Figure 3(a), for x=5 mfp, shows that the buildup factor is generally largest for adipose tissue and smallest for cortical bone in the energy region 1.5>E>5 MeV. This is explained by cortical bone being a high‐$Z_{\text{eq}}$ material due to an appreciable fraction of the medium‐Z element: calcium (z=20, (weight fraction=0.225), whereas adipose tissue is a low‐$Z_{\text{eq}}$ material. Similar results are observed at penetration depths of 15 mfp (Fig. 3(b)) and 40 mfp (Fig. 3(c)) for the energy region E<1.5MeV. So, it can be concluded that the buildup factor depends on chemical composition, in other words, decreases with the increase in $Z_{\text{eq}}$ of the organs/tissues for E<1.5MeV.

However, Fig. 3(a) also shows that in the energy range 1.5<E<5MeV, the buildup factor has about same value for all organs/tissues studied. In other words, the buildup factor is independent of the chemical composition in this energy region, where pair production is a photon interaction process of growing importance. Pair production means that an incident photon is absorbed and a pair of an electron and a positron is created. The positron is rapidly annihilated, creating another pair of 0.511 MeV photons. These secondary gamma photons to some extent compensate for the primary loss of photons. Thus, at the transition from Compton scattering to pair production as the main photon interaction process, there is an energy range where the buildup factor is approximately independent of the chemical composition of the absorbing material, which implies that the buildup factor is independent of $Z_{\text{eq}}$ of the material.

In Fig. 3(b), the penetration depth is larger, 15 mfp, leading to a larger number of secondary photons. An interesting feature is that the buildup factor in this case is independent of the chemical composition for all energies E>1.5 Mev. For the largest penetration depth of 40 mfp (Fig. 3(c)), the buildup factor is independent of the chemical composition in an energy range 1.5<E<3Mev. It follows that the buildup factor is independent of the chemical composition for any penetration depth in the energy range 1.5–3 MeV.

Interestingly, the inset graph of Fig. 3(c) shows that the buildup factor values are maximum for cortical bone and minimum for adipose tissue (the reverse trend compared to Fig. 3(a)) at energies E>3MeV. In other words, for incident photon energy above 3 MeV the buildup factor increases with the increase in $Z_{\text{eq}}$ of the material. A similar trend has been observed for the present human organs/tissues at the penetration depths 15<x≤40 mfp. At energies E>3MeV, pair production is totally dominating, leading to a large number of secondary photons, in particular for high‐Z materials and large penetration depths. Thus, the material with the highest atomic number will have the largest buildup factor, in contrast to the case of low‐Z depicted in the inset graph of Fig. 3(a).

#### B.3 Dependence on the effective atomic number, $Z_{\text{eff}}$

**Figure 4:**
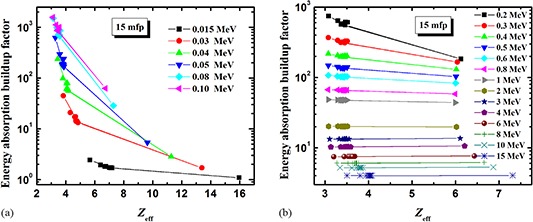
The energy absorption buildup factor as a function of the effective atomic number, $Z_{\text{eff}}$, at 15 mfp for energy range: (a) 0.015–0.1 MeV, and (b) 0.2–15 MeV.

**Figure 5:**
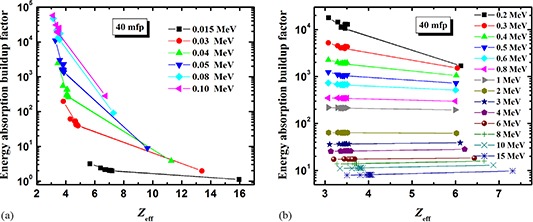
The energy absorption buildup factor as a function of the effective atomic number, $Z_{\text{eff}}$, at 40 mfp for energy range: (a) 0.015–0.1 MeV, and (b) 0.2–15 MeV.

Recently, we have derived a comprehensive and consistent set of formulas for $Z_{\text{eff}}$ valid for all types of materials and for all photon energies greater than 1 keV.^(^ ^5^ ^)^ These formulae have been applied for materials of biological and medical interest.^(^ ^16^ ^–^ ^20^ ^)^ In the present investigation, we have used these formulae to study the behavior of energy absorption buildup factor as a function of $Z_{\text{eff}}$. Figures 4 and 5 show the buildup factor as a function of $Z_{\text{eff}}$, which further substantiates the observations of the preceding section.

Figure 4 shows the energy absorption buildup factor as a function of $Z_{\text{eff}}$ for penetration depth of 15 mfp. At low energies (Fig. 4(a)), the buildup factor is markedly decreased with increasing $Z_{\text{eff}}$. This trend is mainly due to the presence of medium‐ to high‐Z elements (P, S, Cl, K, Fe and Ca). This behavior is due to the fact that in the lower‐energy region, the photoelectric absorption is the most dominant process. So, for a given value of incident photon energy as one moves from the lower to the higher $Z_{\text{eff}}$ side, the photons are more readily absorbed by photoelectric interaction. Hence their lifetime in the materials is small, which results in lowering the value of buildup factor. However, at energies greater than 1 MeV (Fig. 4(b)), the buildup factor is seen to be independent of $Z_{\text{eff}}$. This confirms that the chemical composition of materials does not affect their attenuation properties at higher incident photon energies.

At the larger penetration depth of 40 mfp, the buildup factor follows the general trend and decreases with increasing $Z_{\text{eff}}$ at low energies (Fig. 5(a)), but the behavior is reversed at high energies. For E>3MeV, the buildup factor slowly increases with increased $Z_{\text{eff}}$ (Fig. 5(b)).

#### B.4 Calculation of uncertainty

The geometric progression (GP) fitting seems to reproduce the buildup factors with better accuracy when compared with other available approximations such as Berger, Taylor and three exponential.^(^ ^1^ ^)^ The absolute value of the maximum deviation of buildup factors for water in the GP fitting is within 0.5%–3%, in the three‐exponential approach it is within 0.4%–9.3%, in the Berger approach it is within 0.9%–42.7%, and in the Taylor approximation it is within 0.4%–53.2%.^(^ ^1^ ^)^ Recently, Asano and Sakamoto^(^ ^21^ ^)^ have compared their buildup factors of two typical heavy concretes evaluated by using Monte Carlo simulations code (EGS4) with ANSI/ ANS‐6.4.3 standard reference database, and concluded that there is good agreement between both, except occasional slight differences. These differences may be due to the fact that ANSI/ ANS data is based on the calculations using the moments method^(^ ^22^ ^)^ with parallel beam source and the Monte Carlo code EGS4 with isotropic emission source. Shimizu et al.^(^ ^23^ ^)^ have reported that when using the invariant embedding, GP fitting and Monte Carlo methods agree well for 18 low‐Z materials with small discrepancies. All the materials used in the present study consist of low‐Z materials. For the time being, there are no new reference data for buildup factors. Meanwhile, it should be all right to use the ANSI/ANS data, since the possible discrepancies are expected to be small for the low‐Z materials of the present study.

### C. Tissue equivalent materials

Phantoms are constructed from materials having good tissue equivalence with respect to absorption of ionizing radiation (e.g., gamma and X‐rays). The fundamental advantage of such materials is that they allow the absorbed dose to be determined when information on the energy and nature of the charged particles at the point of interest is incomplete. For a given radiation type and energy, these materials should absorb and scatter radiation to the same extent, within acceptable limits, as irradiated tissue. The chemical compositions of tissue‐equivalent materials were obtained from the literature.^(^ ^10^ ^,^ ^24^ ^)^

To facilitate the formulation of tissue substitutes for a wide range of applications (e.g., dosimetric phantoms, radiographic test objects, dosimeter components, etc.), a procedure has been proposed for photon interactions. This procedure involves the calculation and comparison of $Z_{\text{eff}}$ of the material (to be used as tissue substitute material) with the present human organ/tissues in the extended energy region 1 keV–100 GeV, as described by Manohara et al.^(^ ^5^ ^)^ For photons, $Z_{\text{eff}}$ of the material should match as closely as possible to that of the human organ/tissues to be irradiated.

**Figure 6:**
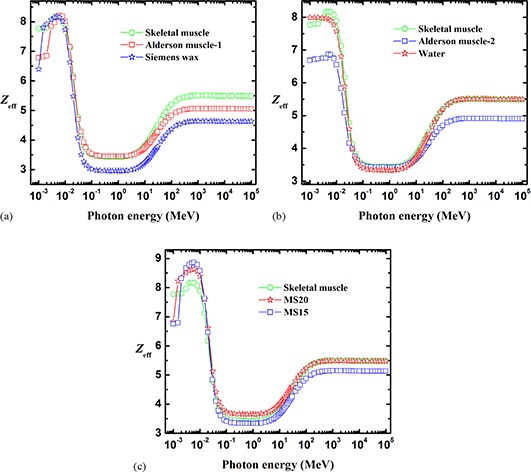
The effective atomic number, $Z_{\text{eff}}$, of skeletal muscle compared with that of: (a) Alderson muscle‐1 and Siemens wax, (b) Alderson muscle‐2 and water, and (c) MS20 and MS15.

The energy dependence of $Z_{\text{eff}}$ for total photon interaction is shown in Fig. 6(a)–6(c) for skeletal muscle and for the tissue‐equivalent materials such as Alderson muscle‐1, Siemens wax, Alderson muscle‐2, water, MS20, and MS15. It is seen that the energy behavior of the effective atomic number of skeletal muscle is qualitatively well described by these tissue‐equivalent materials.

Figure 6(a) shows that Alderson muscle‐1 simulates skeletal muscle particularly well between $5\times 10^{-3}$ and $1.5\times 10^{1}\text{MeV}(5\text{keV}-15\text{MeV})$, and Siemens wax in the smaller energy range from $1.5\times 10^{-3}$ to $8\times 10^{-3}\text{MeV}$ (1.5keV−8keV).

Figure 6(b) shows that water is an good tissue‐equivalent material for skeletal muscle in the wide energy range $3\times 10^{-2}$ to $10\times 10^{1}$ GeV (30 MeV–100GeV). Alderson muscle‐2 is useful at energies from $8\times 10^{-2}$ to $7\times 10^{0}\;\text{MeV}\;(80\;\text{keV}–7\;\text{MeV})$.

Figure 6(c) shows that MS15 is particularly useful for simulating skeletal muscle at energies from $3\times 10^{-2}$ to $9\times 10^{0}\;\text{MeV}\;(30\;\text{keV}–9\;\text{MeV})$. MS20 is useful in the wider energy range from $6\times 10^{1}$ to $10^{5}\;\text{MeV}\;(60\;\text{MeV}–100\;\text{GeV})$, and it slightly overestimates $Z_{\text{eff}}$ between $10^{-1}$ and $10^{1}\;\text{MeV}\;(100\;\text{keV}–10\;\text{MeV})$.

**Figure 7:**
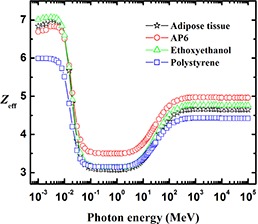
The effective atomic number, $Z_{\text{eff}}$, of adipose tissue compared with that of AP6, ethoxyethanol, and polystyrene.

Figure 7 shows that ethoxyethanol is a good tissue‐equivalent material for adipose tissue in the energy range of 3 keV–80 MeV. Polystyrene is useful in the energy range from $4\times 10^{-2}$ to $4\times 10^{1}\;\text{MeV}\;(40\;\text{keV}–40\;\text{MeV})$, whereas AP6 is useful in the low‐energy range from $8\times 10^{-3}$ to $10\times 10^{-2}\;\text{MeV}\;(8–10\;\text{keV})$.

**Figure 8:**
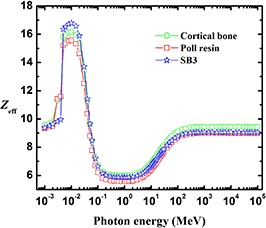
The effective atomic number, $Z_{\text{eff}}$, of cortical bone compared with that of poll resin and SB3.

**Figure 9:**
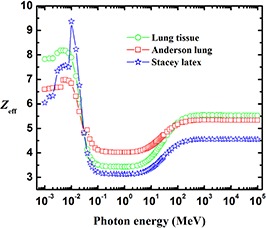
The effective atomic number, $Z_{\text{eff}}$, of lung tissue compared with that of Anderson lung and Stacey latex.

Figure 8 shows that poll resin and SB3 can be used as tissue‐equivalent materials for cortical bone in the energy regions 3–4 keV and 50 keV–5 MeV, respectively. Figure 9 shows that Alderson lung (30 keV) and Stacey latex (at 30 keV and 50 MeV–100 MeV) can be used as phantom materials for lung tissue in the specified energy regions.

## IV. CONCLUSIONS

The GP fitting formula has been successfully applied for the computation of the energy absorption buildup factor of twelve human organs and tissues. The generated energy absorption buildup factor has been studied as a function of photon energy, chemical composition, and effective atomic number.

The chemical composition, $Z_{\text{eq}}$, plays an important role for buildup of gamma photons within the selected human organs/tissues. Below 1.5 MeV and for any penetration depth up to 40 mfp, the energy absorption buildup factor decreases with the increase in $Z_{\text{eq}}$ of the human organ/tissue. Above 3 MeV, the following applies:

- For small penetration depths (less than 15 mfp), the energy absorption buildup factor decreases with increasing $Z_{\text{eq}}$ of the material.
- For a penetration depth of about 15 mfp, the energy absorption buildup factor is independent of $Z_{\text{eq}}$ of the material.
- For large penetration depths (larger than 15 mfp), the energy absorption buildup factor increases with increasing $Z_{\text{eq}}$ of the material.

As a corollary, there is an energy range 1.5<E<3 MeV, where the buildup factor is independent of $Z_{\text{eq}}$ for any penetration depth up to 40 mfp.

Water and MS20 are good tissue‐equivalent materials for skeletal muscle in the extended higher energy range. Similarly, ethoxyethanol and polystyrene are very useful for simulating adipose tissue, and SB3 is useful for cortical bone in the specified energy regions for photon interactions. Other tissue‐equivalent materials are useful in limited energy ranges, as specified.

Gamma rays and X‐rays are extensively used in medical field for diagnosis and treatment of many diseases such as cancer. With proper knowledge of the buildup of photons in human organs and tissues, energy absorption in the human body can be carefully controlled. The present results will also help in estimating safe dose levels for radiotherapy patients, and should prove useful in radiation therapy, diagnostics, and dosimetry.

## References

[1] Harima Y . An historical review and current status of buildup factor calculations and applications. Radiat Phys Chem. 1993;41(4–5):631–72.

[2] American National Standard Institute . Gamma‐ray attenuation coefficients and buildup factors for engineering materials. Report ANSI/ANS‐6.4.3‐1991. La Grange Park, Illinois: American Nuclear Society; 1991.

[3] Ruggieri LP and Sanders CE . Update to ANSI/ANS‐6.4.3‐1991 gamma‐ray buildup factors for high Z engineering materials (Part I). Trans Amer Nucl Soc. 2008;99:618–20.

[4] Ryman JC , Alpan FA , Durani LA , et al. Revision of ANSI/ANS‐6.4.3. Trans Amer Nucl. Soc. 2008;99:613–14.

[5] Manohara SR , Hanagodimath SM , Thind KS , Gerward L . On the effective atomic number: a comprehensive set of formulas for all types of materials and energies above 1 keV. Nucl Instrum Methods Phys Res Section B. 2008;266(18):3906–12.

[6] Gerward L , Guilbert N , Jensen KB , Levring H . X‐ray absorption in matter. Reengineering XCOM. Radiat Phys Chem. 2001;60(1–2):23–24.

[7] Gerward L , Guilbert N , Jensen KB , Levring H . WinXCom – a program for calculating X‐ray attenuation coefficients. Radiat Phys Chem. 2004;71(3–4):653–54.

[8] Kurudirek M and Özdemir Y . Energy absorption and exposure buildup factors for some polymers and tissue substitute materials: photon energy, penetration depth and chemical composition dependence. J Radiol Prot. 2011;31(1):117–28. 21346285 10.1088/0952-4746/31/1/008

[9] Harima Y , Sakamoto Y , Tanaka S , Kawai M . Validity of geometric‐progression formula in approximating gamma‐ray buildup factors. Nucl Sci Eng. 1986;94(1):24–35.

[10] International Commission on Radiation Units and Measurements (ICRU) . Tissue substitutes in radiation dosimetry and measurement. Report No. 44 (ICRU‐44). Bethesda, MD: ICRU; 1989.

[11] Hubbell JH and Seltzer SM . Tables of X‐ray mass attenuation coefficients and mass energy‐absorption coefficients 1 keV to 20 MeV for elements Z = 1 to 92 and 48 additional selected substances of dosimetric interest. NISTIR–5632. Gaithersberg, MD: NIST; 1995.

[12] Harima Y . An approximation of gamma‐ray buildup factors by modified geometrical progression. Nucl Sci Eng. 1983;83(2):299–309.

[13] Maron MJ . Numerical analysis: a practical approach. New York: Macmillan; 1987.

[14] Sidhu GS , Singh PS , Mudahar GS . Energy absorption buildup factor studies in biological samples. Rad Prot Dosim. 1999;86(3):207–16.

[15] Bhandal GS and Singh K . Effective atomic number studies in different biological samples for partial and total photon interactions in the energy region $10^{-3}$ to $10^{-5}\;\text{MeV}$ . Appl Radiat Isot. 1993;44(3):505–10. 8472023 10.1016/0969-8043(93)90161-3

[16] Manohara SR and Hanagodimath SM . Studies on effective atomic numbers and electron densities of essential amino acids in the energy range 1 keV‐100 GeV. Nucl Instrum Methods Phys Res Section B. 2007;258(2):321–28.

[17] Manohara SR and Hanagodimath SM . Effective atomic numbers for photon energy absorption of essential amino acids in the energy range 1 keV to 20 MeV. Nucl Instrum Methods Phys Res Section B. 2007;264(1):9–14.

[18] Manohara SR , Hanagodimath SM , Gerward L . Energy dependence of effective atomic numbers for photon energy absorption and photon interaction: studies of some biological molecules in the energy range 1 keV–20 MeV. Med Phys. 2008;35(1):388–402. 18293593 10.1118/1.2815936

[19] Manohara SR , Hanagodimath SM , Gerward L . Studies on effective atomic number, electron density and kerma for some fatty acids and carbohydrates. Phys Med Biol. 2008;53(20):N377–N386. 18812646 10.1088/0031-9155/53/20/N01

[20] Manohara SR , Hanagodimath SM , Gerward L . The effective atomic numbers of some biomolecules calculated by two methods: a comparative study. Med Phys. 2009;36(1):137–41. 19235382 10.1118/1.3030952

[21] Asano Y and Sakamoto Y . Gamma‐ray buildup factors for heavy concretes. JAEData/Code 2007‐006. Ibaraki‐ken, Japan: Japan Atomic Energy Agency; 2007.

[22] Eisenhauer CM and Simmons GL . Point isotropic gamma‐ray buildup factors in concrete. Nucl Sci Eng. 1975;56:263–70.

[23] Shimizu A , Onda T , Sakamoto Y . Calculation of gamma‐ray buildup factors up to depths of 100 mfp by the method of invariant embedding, (III) generation of an improved data set. J Nucl Sci Technol. 2004;41(4):413–24.

[24] White DR . Tissue substitutes in experimental radiation physics. Med Phys. 1978;5(6):467–79. 366367 10.1118/1.594456

